# The Effects of Repeated Attachment Security Priming on Social Anxiety and Attention Bias: A Randomized Controlled Trial

**DOI:** 10.3390/bs13050420

**Published:** 2023-05-16

**Authors:** Shuang Zhang, Yanqiang Tao, Yunxiang Chen, Peng Zhang, Xiangping Liu

**Affiliations:** 1 Beijing Key Laboratory of Applied Experimental Psychology, National Demonstration Center for Experimental Psychology Education (Beijing Normal University), Faculty of Psychology, Beijing Normal University, Beijing 100875, China; zhshuang525@163.com (S.Z.); psyxuele2020@163.com (Y.T.);; 2 Faculty of Psychology, Beijing Normal University, Beijing 100875, China; 3 Department of Psychology, Tsinghua University, Beijing 100084, China

**Keywords:** social anxiety, attachment security priming, attention bias, undergraduate

## Abstract

Background: Although the clinical utility of attachment security priming has been suggested in recent years, the effect of attachment security priming on social anxiety and its core symptoms (i.e., attention bias) remains unspecified. Therefore, the present study explored the potential effectiveness of repeated attachment security priming in alleviating social anxiety and attention bias among Chinese college students. Methods: Fifty-six college students with high social anxiety were randomly assigned to the attachment security priming group (*n* = 30) or control group (*n* = 26). The priming group completed seven attachment security priming sessions over 2 weeks (every 2 days), and the control group was assigned to a waitlist for 2 weeks. Results: The results revealed that individuals in the priming group reported less social anxiety after 2 weeks of security attachment priming, and those in the control group did not change significantly. The results also showed that there was no significant change in the attention bias of individuals with social anxiety before and after the intervention. Conclusions: Our findings indicate that attachment security priming is a promising alternative intervention option for social anxiety. The potential clinical implications of security attachment priming are discussed.

## 1. Introduction

Social anxiety disorder (SAD, also known as social phobia) is one of the earliest and most common disorders among all anxiety disorders [1]. The core symptoms of individuals with SAD are clear and persistent tension and fear, even avoidance behavior, when they are exposed to social situations that can be observed and evaluated by others [2]. Social anxiety begins to rise in adolescence and reaches its peak among college students [3]. Notably, up to 25.8% of undergraduate students report clinical levels of social anxiety [4]. Although some individuals did not meet the diagnostic criteria for SAD, they showed similar, mild cognitive and behavioral symptoms [5]. Social anxiety (SA) involves less participation in social activities, less public expression, and less access to social resources, which in turn, leads to impaired social function [6]. Due to their inability to express themselves properly in class or the workplace and fear of speaking, talking, or even eating in public, this increases the risk of many psychological problems, such as sleep disorders, substance addiction, depression, and suicidal ideation [7,8]. In short, social anxiety results in interpersonal difficulties and damages mental health.

To provide ideas for the treatment of social anxiety, researchers have developed pharmacological and psychological interventions. Specifically, a series of drugs (i.e., selective serotonin reuptake inhibitors) aimed at the physiological factors of social anxiety (including heredity, brain nerve abnormalities, etc.) are used to treat social anxiety. However, in most cases, the response rate of the above common drugs is not successful (40~60%) [9]. Additionally, the high costs and large side effects (e.g., withdrawal symptoms and relapse) hinder the treatment of SA. Compared with drug therapy, psychotherapy shows broader application prospects [10]. In many psychological treatment conditions, cognitive behavioral therapy (CBT) has been proven to have a greater effect than other psychotherapies, such as mindfulness-based stress reduction [11], psychodynamic psychotherapy [12], and interpersonal psychotherapy [13]. CBT can effectively change maladaptive beliefs, reduce the likelihood of overestimating social threats, and improve the quality of life of patients with SAD [14,15]. However, it is notable that some non-specific factors (i.e., the therapeutic alliance, the therapist’s competence, and adherence to the treatment protocols) may account for a large portion of its efficacy. Additionally, a recent meta-analysis indicated that the recovery rate of SAD after CBT treatment is only 35%, which is lower than 54% of other anxiety disorders [16]. More importantly, even with a combination of psychotherapy (i.e., CBT) and pharmacotherapy, many patients fail to respond or relapse [9]. Additionally, given the core characteristics of social anxiety, the fear of negative comments from doctors and therapists can hinder seeking treatment [17] and lead to a high dropout rate from treatment [18]. Therefore, it is vital to develop innovative, effective, and accessible interventions to alleviate social anxiety.

Attachment theory is highlighted as an important theoretical framework for understanding SA [19]. The core assumption of attachment theory is that an attachment figure (usually parents or caregivers) is a “secure base” for an infant to explore the environment and is a safe haven to which to return for reassurance [20]. Attachment theory proposes that the interaction pattern between infants and caregivers will gradually be internalized into a relatively stable internal working model (IWM) [21]. The IWM, as a deep cognitive structure, usually automatically instructs individuals’ core beliefs and expectations in interpersonal relationships. Sensitive and reactive interaction contributes to a positive IWM and secure attachment orientation, whereas insensitive and unavailable parenting contributes to a negative IWM and insecure attachment orientation [22]. Ainsworth et al. pioneered the use of the Strange Situation Procedure to determine attachment patterns, which can be divided into secure, anxious-ambivalent, and avoidant styles [23]. Attachment orientations are conceptualized along two independent dimensions: Avoidance of intimacy and anxiety regarding abandonment [24]. Those who score low on both dimensions are considered as secure attachment. Insecure attachment is closely associated with a variety of mental health issues, such as depression, anorexia, and substance use [25]. A theory of attachment style and psychopathology suggests that when an anxiously attached individual is vigilant to threats and is exposed to a chronically threatening environment, this can lead to the development of an anxiety disorder [26]. Consistently, the empirical studies support the above theoretical propositions. For example, research has shown a significant correlation between social anxiety and insecure attachment in both general population samples [27] and clinical samples [28]. Individuals with insecure attachment (i.e., attachment anxiety and attachment avoidance) showed higher levels of social anxiety [29]. Insecure attachment can significantly predict social anxiety even after controlling for social comparison, submissiveness, and depression [30]. Conversely, individuals with secure attachment experience less social anxiety in social interaction. The abovementioned studies indicated that attachment style is closely related to social anxiety, and insecure attachment individuals are more likely to show social anxiety than secure attachment individuals.

Fortunately, security attachment is malleable, and a sense of security can be temporarily acquired by presenting secure attachment-related stimuli (such as pictures, words, and story situations) or by asking participants to imagine (recall) a secure attachment-related experience [31]. Both implicit and explicit priming procedures can effectively enhance attachment security [32]. Growing research has documented positive outcomes through priming attachment security (i.e., presenting stimuli elicit security attachment), such as more positive self-views [33], enhanced empathy [34], and reduced rumination [35].

Encouragingly, researchers have shown that mood disorders may be mitigated through interventions that improve secure attachment [32,36]. Emerging evidence supports that repeated security priming could reduce anxiety and depression in nonclinical samples and clinical samples [37,38]. These findings suggest that increasing attachment security may help in relieving emotional pain. However, to date, little research has examined the efficiency of repeated attachment security priming on social anxiety specifically. Considering the low cost and easy availability of security attachment priming, it can be used as a complementary intervention to address low response rates and fear of seeking help.

To date, few studies have focused on the effects of psychosocial treatments of SA on attention bias. The cognitive model emphasizes that attention bias is a key factor in the occurrence and maintenance of social anxiety [39]. Empirical studies have also demonstrated that compared to healthy controls, patients with SAD are more vigilant to threat stimuli or more avoidance to threatening stimuli [40,41,42]. Mogg et al. propose the vigilance–avoidance hypothesis, which suggests that SA patients are initially hypervigilant for threats, and then with the increase in anxiety, they would immediately enter the avoidance stage to reduce anxiety experience [43]. Attention bias is an important feature of social anxiety, and the alleviation of social anxiety may be accompanied by a decrease in attention bias [44]. Limited studies provide evidence that attention bias toward a threat is significantly reduced after the course of CBT [45,46]. These promising findings indicate that attention bias may be diminished due to the reduction in social anxiety. However, the effect of other SA interventions (i.e., attachment security priming) on attention bias is unclear. Clarifying whether attention bias can be changed by intervention could help us in deeply understanding the role of attention bias in social anxiety.

Considered together, this study aimed to examine whether repeated attachment security priming is a potentially effective intervention for social anxiety and attention bias in Chinese undergraduates. Given the chronic course and characteristics of social anxiety, repeated attachment security priming was used in this study, which revealed greater and more persistent benefits than single priming [47]. Based on the theoretical constructs and previous studies, we hypothesize that the priming group, compared with the unprimed control group, would experience lower social anxiety after repeated attachment security. Furthermore, according to the vigilance–avoidance model of attention, it is expected that participants will initially be more vigilant for the threatening stimuli than the neutral or positive stimuli, but will exhibit avoidance of threatening stimuli. We also expect that attention bias (vigilance or avoidance) could be reduced by repeated attachment security priming.

## 2. Method

### 2.1. Study Design

A parallel randomized controlled design was employed in this study. It complies with the Consolidated Standards of Reporting Trials (CONSORT) checklist to explore the effectiveness of repeated attachment security priming in alleviating social anxiety and attention bias among Chinese college students.

### 2.2. Participants

**Figure 1:**
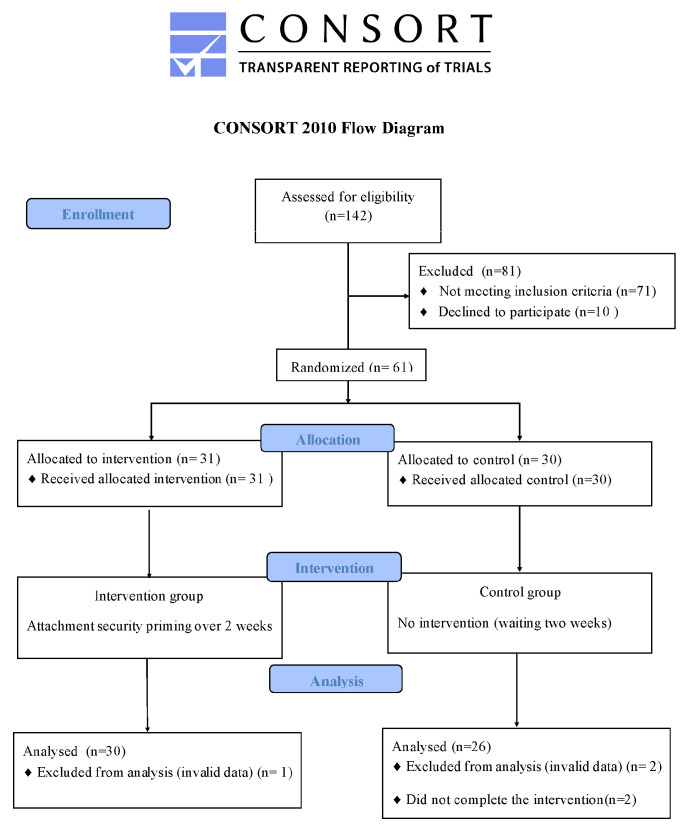
Flowchart of participants in the randomized controlled trial.

Undergraduate students (*n* = 142) were recruited via online advertising and campus posters from one university in Gansu Province, China. Participants provided informed consent to participate and completed the questionnaires. Participants whose scores were higher than the median in the Interaction Anxiety Scale (IAS) were invited to participate in the study [48]. A total of 61 college students participated in this study, the researchers randomly divided participants into a priming group (*n* = 31) and a control group (*n* = 30) using a Microsoft Excel random number generator formula. Among them, two participants did not complete the experimental task, and another two participants were eliminated from data analyses due to their invalid data in the control group. One participant was eliminated from data analyses due to their invalid data in the priming group. Therefore, the final sample consisted of 30 participants in the priming group (24 females) and 26 participants in the control group (16 females). Participants in the control group were first assigned to the waitlist and received the same priming as the training group after the waitlist period (2 weeks). Figure 1 shows the process of recruitment of the participants. All participants were right-handed and had normal or corrected-to-normal vision without an apparent physical or mental disorder. They were compensated for their time at a rate of CNY ¥60. A prior power calculation was calculated using GPOWER 3.1. With an alpha of 0.05 and a power of 0.80, the projected sample size needed for a medium effect (*f* = 0.25) is 34 participants, with approximately 17 participants for each group. Therefore, our sample of 56 participants was adequate. The study was approved by the local Research Ethics Committee (IRB Number: 201912230086).

### 2.3. Questionnaires

#### 2.3.1. Experience of Close Relationship-Short, ECR-S

The Chinese version of the ECR-S is based on the English version of the ECR [24,49]. It consists of 18 items on two dimensions, which include attachment anxiety (e.g., “I worry about being abandoned”) and attachment avoidance (e.g., “I get uncomfortable when someone wants to be very close to me”). Participants were asked to rate each statement on a 7-point Likert scale, ranging from 1 (strongly disagree) to 7 (strongly agree). The mean score of each subscale was calculated, with a higher score indicating stronger attachment anxiety or avoidance. The internal consistency reliability (as measured by Cronbach’s α) was 0.828 for the attachment anxiety scale and 0.819 for the attachment avoidance scale in this study.

#### 2.3.2. Interaction Anxiety Scale, IAS

The Chinese version of the IAS is based on the English version of the IAS [48,50]. It consists of 15 items, which were used to assess the subjective feelings of the participants who were detached from the social scene (e.g., “Parties often make me feel anxious and uncomfortable”). Participants were asked to rate each statement on a 5-point Likert scale, ranging from 1 (disagree strongly) to 5 (agree strongly). Higher scores indicated higher levels of social anxiety. The internal consistency reliability (as measured by Cronbach’s α) in this study was 0.880.

### 2.4. Procedures

Participants were invited to come to the laboratory, and after providing informed consent, they completed spatial cueing tasks. To disguise the actual purpose, participants were told they would practice meditation seven times over 2 weeks (once every other day). Research suggests that meditating on close relationships may facilitate a sense of state-level security [51]. Participants finished the first attachment security priming in the laboratory according to the recorded instructions. Then, the research assistant was responsible for playing the recording and keeping the scene quiet during the entire process. To ensure that participants performed the imagination task every 2 days, they were asked to fill in a log sheet detailing when and where they accomplished each task. All participants returned to the laboratory 2 weeks later and completed the post-test questionnaire and spatial cueing task. Finally, participants were debriefed, paid, and thanked for participating.

### 2.5. Attachment Security Priming

The guided imagination task was used, which was adapted from previous studies [52,53]. The priming materials for secure attachment were audio recorded by psychological professionals. The recording lasted 17 min. Examples of instructions are as follows:

Please take a few deep breaths to relax yourself. Imagine yourself in secure relationships and being loved and supported by important others. He/she can be your family, friends, or a lover. You trust that they truly love you and will not abandon you or try to distance themselves from you. You are interdependent and intimate. You enjoy the feeling of connection. You feel safe, comfortable, and fulfilled.

### 2.6. Spatial Cueing Task

Face stimuli were selected from the Chinese Affective Face Picture system (CAFPS; [54], which consisted of 16 face pictures of 12 individuals (6 males). The faces portrayed three different emotional expressions: Disgust, happy, and neutral. The intensities of the images were as follows: 4 happy (*M* = 6.1, *SD* = 1.3), 4 disgust (*M* = 6.3, *SD* = 0.7), and 8 neutral (*M* = 5.6, *SD* = 0.2). There was no significant difference in the intensities of the three emotions (*F*(2, 23) = 1.523, *p* = 0.241). All face images were edited with Photoshop 7.01 to be the same size (10.8 cm × 12.7 cm), brightness, black, and white. The tasks were controlled by the E-Prime 2.0 software, and the experiment was displayed on a 21-inch monitor with a resolution of 1920 × 1080 pixels. The experiment was conducted in a dimly lit laboratory with a computer screen at a viewing distance of 40 cm.

Each trial began with a central, white fixation cross presented for 500 ms. Then, two gray boxes were presented for 500 ms to the left and right of a white central fixation cross (which remained on the screen throughout the trial). Thereafter, a face cue randomly appeared to the left or right of the gray box (4 cm × 5 cm) for 500 ms, and 500 ms later, a target (the letter E or F, 4 cm × 5 cm) was presented. Participants will be presented with three types of facial expressions (disgust, happiness, neutral) randomly. Immediately after the presentation of each facial expression, a letter “E” or “F” will appear in the same location as the facial expression. If the letter is “E”, participants are instructed to click the left mouse button. If the letter is “F”, participants are instructed to click the right mouse button. If the response was correct, the screen displayed feedback with a smiley face and the subtitle “great”, and if the response was wrong, the screen feedback displayed feedback with a crying face and the subtitle “keep trying”, the feedback was presented for 500 ms, and then the next trial began (see Figure 1). Each face cue and target appeared equally often in the left and right locations.

**Figure 2:**
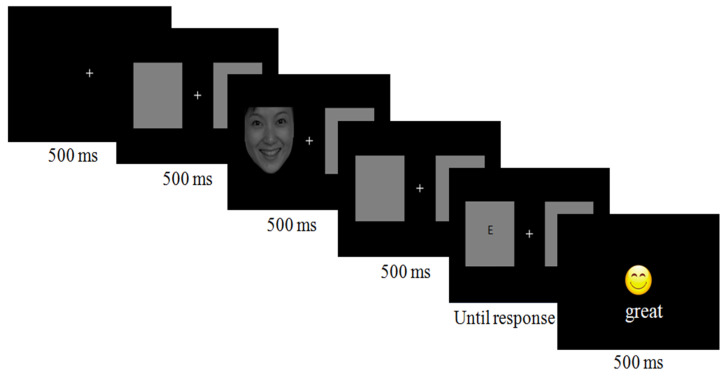
Schematic diagram of spatial cueing task.

Each participant was given three practice trials, and when the accuracy of the practice trial exceeded 80%, the experimental trials began. The practice task included 4 neutral faces (repeated 3 times) that were not included in the experimental block. The experimental task included 12 faces (4 neutral, 4 disgust, and 4 happy), which were repeated 4 times, and the probability of appearing on the screen left or right was equal. Therefore, the experimental trials consisted of 96 (12 × 4 × 2) trials, which were randomly divided into 4 blocks, and each block included 24 trials. The reaction times (RTs) and accuracies were recorded for each trial. A flowchart of the procedure can be seen in Figure 2. Overall, the task lasted approximately 15 min.

The target that appeared in the same spatial location as the face cue was the valid position while the target that appeared in the opposite location to the face cue was the invalid position. In valid trials, if the mean RT for threat cues (i.e., disgusted face) was less than the mean RT for neutral cues, this indicated attention hypervigilance. In invalid trials, the mean RT on threat cues was more than the mean RT on neutral cues, which indicated attentional difficulty in disengagement. For the same emotional cue, the difference in the RT between the valid trials and the invalid trials was an indicator of attention avoidance [55].

### 2.7. Data Analysis

Analyses were conducted using SPSS version 20.0.

The threshold for statistical significance was set at *p* < 0.05 for all analyses.

Participant characteristics were analyzed using descriptive statistics and *t*-tests for group comparisons. Repeated measures ANOVA was used to assess the effects of group (priming group and control group) and time (pre-test and post-test) on social anxiety and attention bias. Simple effects analyses were used to make pre- and post-test comparisons and between-group comparisons.

According to previous research [56], reaction times were excluded with errors and outliers (high outliers > 1500 ms and low outliers < 200 ms). In total, 3.7% of the data were eliminated.

## 3. Results

### 3.1. Descriptive Statistics

Descriptive statistics are presented in Table 1. Participants assigned to each group did not significantly differ in their demographic characteristics.

### 3.2. Social Anxiety

**Figure 3:**
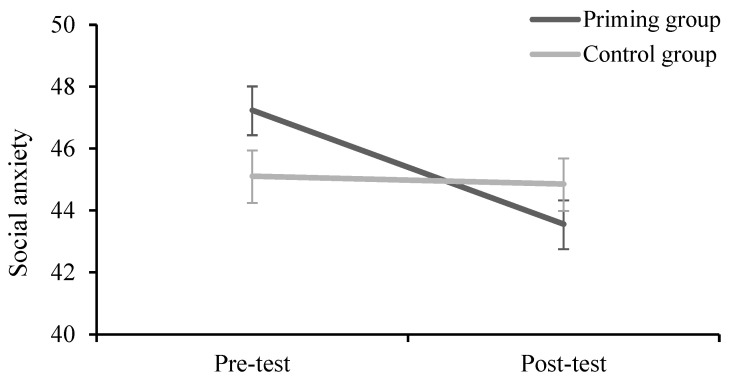
Social anxiety scores in priming and control conditions (error bars represent SEs).

A two (group: Priming, control) × two (time: Pre-test, post-test) repeated-measures ANOVA was conducted with IAS as the dependent variable. The results showed that there was no significant main effect of time, *F*(1,54) = 0.59, *p* = 0.44, *η_p_*^2^ = 0.011, or group, *F*(1,54) = 0.04, *p* = 0.85, *η_p_*^2^ = 0.001. The interaction between group condition and time was significant, *F*(1,54) = 4.30, *p* = 0.043, *η_p_*^2^ = 0.076 (see Figure 3).

The simple effects analysis revealed that IAS scores were significantly lower at the post-test (*M* = 43.50) than at the pre-test (*M* = 47.03, *p* < 0.001) for the priming group, but there was no significant difference between pre- and post-test for the control group (*p* > 0.05). There was also no significant difference between the priming group and the control group in pre- and post-tests (*p* > 0.05).

### 3.3. Attention Bias

Mean reaction times by condition are shown in Table 2.

A two (group: Priming, control) × two (time: Pre-test, post-test) × two (validity: Valid, invalid) × three (emotion: Happy, disgust, neutral) repeated-measures ANOVA was conducted with RTs as the dependent variable. The results showed that there was a significant main effect of trial type, *F*(1,54) = 5.664, *p* < 0.05, *η_p_*^2^ = 0.095, and the RTs of invalid trials (*M* = 559.15) were significantly lower than the RTs of valid trials (*M* = 530.24). The main effect of emotion was also significant, *F*(1,54) = 12. 477, *p* < 0.001, *η_p_*^2^ = 0.188, and the RTs of happy cues (*M* = 559.15) were significantly lower than the RTs of neutral cues (*M* = 545.00) and disgust cues (*M* = 535.96). The interaction between emotion and time was significant, *F*(1,54) = 3.566, *p* < 0.05, *η_p_*^2^ = 0.062. The simple effects analysis revealed that the RTs of disgust cues were significantly faster post-test (*M* = 539.14) than pre-test (*M* = 550.87) (*p* < 0.05), but there was no significant difference between the pre- and post-tests (*p* = 0.71). No other main effects or interactions were significant (all *F*s <2.118, *p*s > 0.05, *η_p_*^2^s < 0.038).

## 4. Discussion

Although the psychological benefits of security attachment priming have been extensively researched, this is the first study to examine whether repeated attachment security priming is a potentially effective priming for social anxiety and attention bias. The current study proposes that repeated secure priming may contribute to relieving social anxiety and attention bias. The results for social anxiety, measured by IAS, supported our hypotheses. Participants in the repeated secure priming group showed diminished social anxiety, whereas the participants in the control group did not show this change over time. The results for attention bias, measured by the spatial cueing task, did not support our hypotheses. Attention bias did not change significantly before and after secure priming, regardless of the priming group or control group.

Although preliminary, the findings reveal that secure attachment priming is a promising alternative intervention option for social anxiety. Past questionnaire studies provide indirect evidence for the relationship between attachment and social anxiety, while our experimental studies provide direct evidence for this relationship. This indicates that the attachment system plays an important role in the induction and maintenance of social anxiety. The results support an integrated theory of understanding social anxiety based on an internal-working model, which involves both evolutionary and cognitive theories [19]. According to dominant cognitive models [39], individuals with social anxiety have negative cognitive biases about the self and others. They view the self as inferior and undesirable and the other as rejective; therefore, they need to be constantly vigilant and strictly self-monitored to avoid harm. However, repeated security priming can increase positive appraisals of the self and relationships [57] and reduce rumination [35]. The change in cognitive schema caused by repeated secure attachment training is a key factor in alleviating social anxiety [58]. Previous intervention studies have found that repeatedly priming attachment security improves anxious moods [37] and depressive symptoms [59]. Extending previous clinical intervention studies, our study further shows that repeated security attachment priming (seven times across 2 weeks) can effectively alleviate social anxiety in this study. The operation of the secure attachment system can improve social skills, increase positive emotions, and promote a sense of security, which will help in alleviating social anxiety.

As revealed in the analysis of attention bias, 2 weeks of attachment security priming had a significant effect on social anxiety but not attention bias. Contrary to our hypothesis, we failed to find that repeated attachment security priming reduced attention bias. Explanations for this result can only be speculative.

First, the results suggested that the social anxiety participants in this study failed to show attention bias, regardless of attentional avoidance or attentional vigilance. According to previous findings, SA may be characterized by an attention bias for emotional faces [60]. They show clear attention vigilance–avoidance to the social stimulus [41]. First, sampling bias may be a possible explanation. To ensure the effectiveness of the intervention, participants were asked to come to the lab for the first attachment security priming. However, individuals with social anxiety tend to avoid social activities, especially in unfamiliar social situations [61]. Moreover, the intervention in this study lasted for a relatively long time (2 weeks), which may require considerable effort for people with social anxiety to fulfill. This might be due to the fact that some participants did not score very high on social anxiety, in return, they did not show clear attentional vigilance or attentional avoidance. Second, the absence of attention bias in SAD was also found in several studies [62,63], which questions the important role of attention bias in social anxiety. Perhaps attention bias is not the main pathogenic factor of social anxiety, and the development of SAD may occur through other pathways [64].

Another possible explanation might be that the repeated secure attachment priming in this study may not be sufficient to change attention bias in adults. However, a CBT study found that attention bias toward threats showed a significant reduction from pre- to post-treatment in children [65], which implies that there may be an age difference that should be noticed. Previous studies have shown that attention bias training, which directly improves attention bias, has a certain effect on changes in attentional patterns, while another cognitive training has difficulty affecting automatic attentional patterns [63,66]. Past studies yielded inconsistent findings, partly due to neglecting moderating variables, such as personality vulnerability factors, intervention methods, etc. Although interest in security attachment priming as an intervention technique is growing, extant findings suggest that cultivating attachment security can be challenging due to attention bias. Given the lack of research in this area, further research is warranted to test the effect of secure attachment priming on the underlying cognitive process (i.e., attention bias).

Overall, this study provides novel insight into interventions for social anxiety. Compared with other psychological interventions for social anxiety, the secure attachment may be more effective. Cognitive behavioral therapy needs to bear the costs of psychological counselors, and attention bias training and interpretation bias training need computers as training carriers. Moreover, given the core feature of social anxiety, people with the condition fear negative evaluations and avoid social occasions. This makes them more reluctant to seek professional help and treatment [67]. However, secure attachment can be implemented through stories, recordings, and pictures, which is more economical and easier to administer. Specifically, these attachment security priming materials can be stored in a mobile phone, written as notes, or decorated in the environment as pictures. For instance, in a field setting, Charles-Sire et al. found that participants donated more blood when they were exposed to t-shirts with security-related words (e.g., loving) than when they were exposed to t-shirts with neutral words (e.g., donating) [68]. Future studies may focus more on the effects of attachment security priming outside of the laboratory.

### Limitations

Similar to any other study, this study is not free from limitations. A main limitation of the present study is the representativeness of samples. Following past research [69,70], we selected college students with elevated social anxiety above the median as the participants. Descriptive statistics showed that the social anxiety scores of the participants in this study (*M* = 45.18) were higher than the average level of the Chinese college student sample (*M* = 38.78) [50]. Although applying median splits is acceptable [71], some participants reported relatively low levels of social anxiety, which may limit the representativeness of the sample. Additionally, the current study only used a self-reported questionnaire as an indicator to assess social anxiety. Future studies can further verify the results by using multiple assessment methods (e.g., behavioral observations and physiological indicators) and different samples (e.g., clinical samples). Noting that the sample used in this study was not a clinical population, caution should be exercised when generalizing the results. Future research can consider conducting robust clinical trials to examine the applicability and effectiveness of the intervention effects reported in this study.

The second limitation is that the control group in this study was a waiting group without priming. The advantage of the unprimed control group is that it allows us to compare the priming effects with the naturalistic growth control group [53]. The downside, however, is that we cannot exclude potentially confounding variables. For instance, positive emotions can produce effects similar to secure attachment [72], and the present study fails to rule out the possibility that positive emotions might produce equivalent effects. Future studies can test the results of this study by using different control conditions, such as a positive emotion control group and a neutral priming group. In this way, it can be clarified whether the positive changes shown by the participants are due to security attachment priming rather than other mixed factors.

Finally, there is a lack of follow-up studies to test the lasting effects of repeated attachment security priming. Studies have found that the effects of attachment security priming can last for a day or even weeks [47,57]. Future research could continue to track the lasting effects of repeated attachment security priming on social anxiety.

## 5. Conclusions

The current study is the first study to indicate that repeated attachment security priming (in the laboratory and via recording) has a beneficial effect on social anxiety. While the findings require replication in a clinical sample, this study offers preliminary evidence that repeated secure priming is a promising approach to alleviate social anxiety among young adults. Attachment security priming is self-helpful, convenient, and easy to operate and can be considered as an alternative or complementary treatment for alleviating social anxiety. Moreover, the evidence adds to the understanding of the effects of security priming and the significance of the clinical practice.

## Figures and Tables

**Table 1:** Descriptive statistics for participants.

	Priming Group (*n* = 30)		Control Group (*n* = 26)		*T*	*p*
	*M*	*SD*	*M*	*SD*		
Age	22.10	4.33	21.73	2.34	0.388	0.699
Pre-IAS	47.03	9.26	45.34	7.60	1.704	0.094
ECR-AV	35.0	10.0	31.0	9.3	1.552	0.127
ECR-AN	29.2	11.0	31.0	8.0	−0.693	0.491

**Table 2:** Comparison of the RTs (ms) between the priming group and the control group.

Time	Cue Validity	Cue Emotion	Priming Group (*n* = 30)		Control Group (*n* = 26)	
			*M*	*SD*	*M*	*SD*
Pre-test	Valid	Happy	564.63	104.82	569.00	92.27
		Neutral	568.07	100.55	569.46	101.52
		Disgust	565.80	110.93	565.62	83.94
	Invalid	Happy	553.07	93.00	554.51	105.35
		Neutral	543.23	98.52	552.68	84.10
		Disgust	546.52	98.07	557.27	87.25
Post-test	Valid	Happy	538.37	112.19	527.67	79.10
		Neutral	538.97	125.51	548.85	88.28
		Disgust	534.17	113.43	537.88	89.22
	Invalid	Happy	525.03	108.48	523.94	89.89
		Neutral	512.61	95.06	535.54	84.96
		Disgust	514.76	116.58	525.07	104.96

## Data Availability

Data are available upon request from the first author.
